# RGmatch: matching genomic regions to proximal genes in omics data integration

**DOI:** 10.1186/s12859-016-1293-1

**Published:** 2016-11-22

**Authors:** Pedro Furió-Tarí, Ana Conesa, Sonia Tarazona

**Affiliations:** 1 Genomics of Gene Expression Laboratory, Gene Expression and Epigenomics Program, Centro de Investigación Príncipe Felipe, Eduardo Primo Yúfera 3, 46012 Valencia, Spain; 2 Microbiology and Cell Science Department, Institute of Food and Agricultural Sciences, University of Florida, Gainesville, FL 32603 USA; 3 Department of Applied Statistics, Operations Research and Quality, Universidad Politécnica de Valencia, Camí de Vera, 46022 Valencia, Spain

**Keywords:** Associations, Gene, Genomic region, Peak, Omics integration, NGS

## Abstract

**Background:**

The integrative analysis of multiple genomics data often requires that genome coordinates-based signals have to be associated with proximal genes. The relative location of a genomic region with respect to the gene (gene area) is important for functional data interpretation; hence algorithms that match regions to genes should be able to deliver insight into this information.

**Results:**

In this work we review the tools that are publicly available for making region-to-gene associations. We also present a novel method, RGmatch, a flexible and easy-to-use Python tool that computes associations either at the gene, transcript, or exon level, applying a set of rules to annotate each region-gene association with the region location within the gene. RGmatch can be applied to any organism as long as genome annotation is available. Furthermore, we qualitatively and quantitatively compare RGmatch to other tools.

**Conclusions:**

RGmatch simplifies the association of a genomic region with its closest gene. At the same time, it is a powerful tool because the rules used to annotate these associations are very easy to modify according to the researcher’s specific interests. Some important differences between RGmatch and other similar tools already in existence are RGmatch’s flexibility, its wide range of user options, compatibility with any annotatable organism, and its comprehensive and user-friendly output.

**Electronic supplementary material:**

The online version of this article (doi:10.1186/s12859-016-1293-1) contains supplementary material, which is available to authorized users.

## Background

The flourishing of sequencing functional genomics assays has popularized the analysis of different chromatin features to understand regulatory aspects of gene expression. These assays measure, for example, the binding of transcription factors or histone modifications at chromosomal locations (chromatin immune precipitation sequencing; ChIP-seq), DNA methylation events (different types of Methyl-seq), or chromatin accessibility (DNase I hypersensitive sites sequencing or Assay for Transposase-Accessible Chromatin with high-throughput sequencing; DNase-seq or ATAC-seq). In all cases, analysis of these data returns potentially functional regions, defined by genomic coordinates, which must then be related to proximal genes in order to gain any biological meaning. How these regions regulate nearby genes depends on the type of experiment. For example, the transcription factor binding sites predicted using ChIP-seq experiments may be expected to be located in the transcription start site (TSS) and promoter regions of the gene being regulated or in distal enhancers depending whether they are cell-type specific or not, and users might want to have control of what association is relevant in their experiment. In the case of open chromatin sites obtained from DNase-seq experiments, the functional interpretation may differ depending if they are in a promoter, intronic, or downstream gene regions. Therefore, it is not only important to associate genomic regions to the closest gene, but also to identify the specific area of the gene where the region is located (the promoter, first exon, an intron, downstream, etc.) [1–5]. The solution to this problem is not straightforward because it depends on the isoform of the gene being considered. In addition, regions may span multiple areas of the same gene (i.e. the TSS and first exon) or fall at overlapping genes. Moreover, regions at intergenic locations can be associated with upstream or downstream areas of different genes, and therefore a set of rules has to be established to decide which association should be kept.

Because current sequencing technologies predict thousands or even millions of genomic regions that must be mapped to other genomic locations such as genes or transcripts in order to perform integration studies, a computational algorithm is required to match these genomic regions to proximal features (e.g. genes). Moreover, it must take the considerations listed above into account, provide users flexibility to set the association criteria, and be easily integrated with broader analysis pipelines. Although there is an increasing need for such algorithms, as far as we know, there are very few publicly-available tools which can perform this task. One such tool is part of the HOMER suite [6], which matches each genomic region to the closest transcript and returns the area of the transcript overlapped by the midpoint of the region. This tool can be used with custom annotations, but other information like the overlapping of CpG islands, repeat elements, etc., is only returned for supported species. GREAT [7] is a web tool for predicting cis-regulatory regions which takes into account not only nearby genes, but also distal binding events. However, the main drawback of GREAT is its lack of support for species other than human, mouse, and zebrafish. CisGenome [8] is one of the first tools that appeared to deal with ChIP-seq data. Among other utilities, it associates regions to proximal genes but does not provide the location of the region within the gene. This tool can either be used via a graphical interface in Windows operating systems or by command line in OSX and Linux. Seq2pathway [9] and ChIPseeker [10] are two different R packages that also contain functions for associating genomic regions with genes and annotate the location of the region within the gene. Seq2pathway follows a similar approach to GREAT but its main limitation is, again, that it only supports two species (human and mouse). In contrast, ChIPseeker is a more complete tool that supports any species, and which associates regions with the closest gene in a similar way to HOMER.

In this work we review the main characteristics and drawbacks of some of these tools and present a novel algorithm, RGmatch, to associate genomic regions with proximal features whilst maintaining the flexibility for researchers to set specific match criteria. RGmatch is implemented in Python so it can either be used as a standalone application or incorporated into any omics analysis pipeline. One advantage of RGmatch is its ability to return associations at the gene, transcript, or exon level. The user can deal with the problem of genomic regions overlapping more than one area of a gene (e.g. both the TSS and first exon), by instructing the algorithm to report all the overlapped gene areas (by choosing the exon aggregation level) or by reporting only one association per transcript or per gene, based on a pre-established set of rules. Importantly, these rules, as well as the width of the TSS, promoter, transcription termination site (TTS), or upstream areas, can be modified to meet the researcher’s needs.

### Methods

**Fig. 1:**
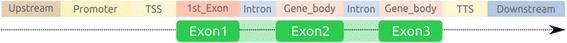
Definition of the areas of a gene used by the RGmatch algorithm

RGmatch is rule-based Python software designed to associate genomic regions to genes, transcripts, or exons that also reports the area of the gene where the region overlaps. It requires two essential input files: the genome annotation in GTF format (http://www.ensembl.org/info/website/upload/gff.html) and the chromatin locations of the genomic regions in BED format (https://genome.ucsc.edu/FAQ/FAQformat.html#format1). RGmatch associates each genomic region with the closest gene (or genes in case of ties resulting from the set of rules used). The distance is computed as the number of bases from the region midpoint to the transcript TSS or TTS. To annotate the area of the transcript where the region falls, we defined eight default disjoint areas (Fig. 1): TSS, TTS, 1st EXON, PROMOTER, INTRON, GENE BODY, UPSTREAM, and DOWNSTREAM. These areas are defined as follows:

- TSS: Intergenic area adjacent to the TSS point of the gene with a length of *t* (200 bp by default).
- Promoter: Intergenic area upstream of the TSS with a length of *p* (1300 bp by default).
- Upstream: Intergenic area upstream of the promoter area, hence more than *t + p*bp from the TSS point of the gene. This length is limited by the maximum distance, *q*, allowed by the user, to associate a region with a gene (10 kbp by default).
- 1st_Exon: The whole of the first exon of the gene.
- Intron: The whole area between two consecutive exons of a gene.
- Gene_body: The whole area of any exon other than the first exon of the gene.
- TTS: Intergenic area adjacent to the TTS point of the gene with a length of *s* (0 bp by default).
- Downstream: The intergenic area downstream of the TTS area, hence more than *s*bp from the TTS point of the gene. The length of this area is limited by the maximum distance, *q*, allowed by the user, between the region and the gene (10 kbp by default).

**Fig. 2:**
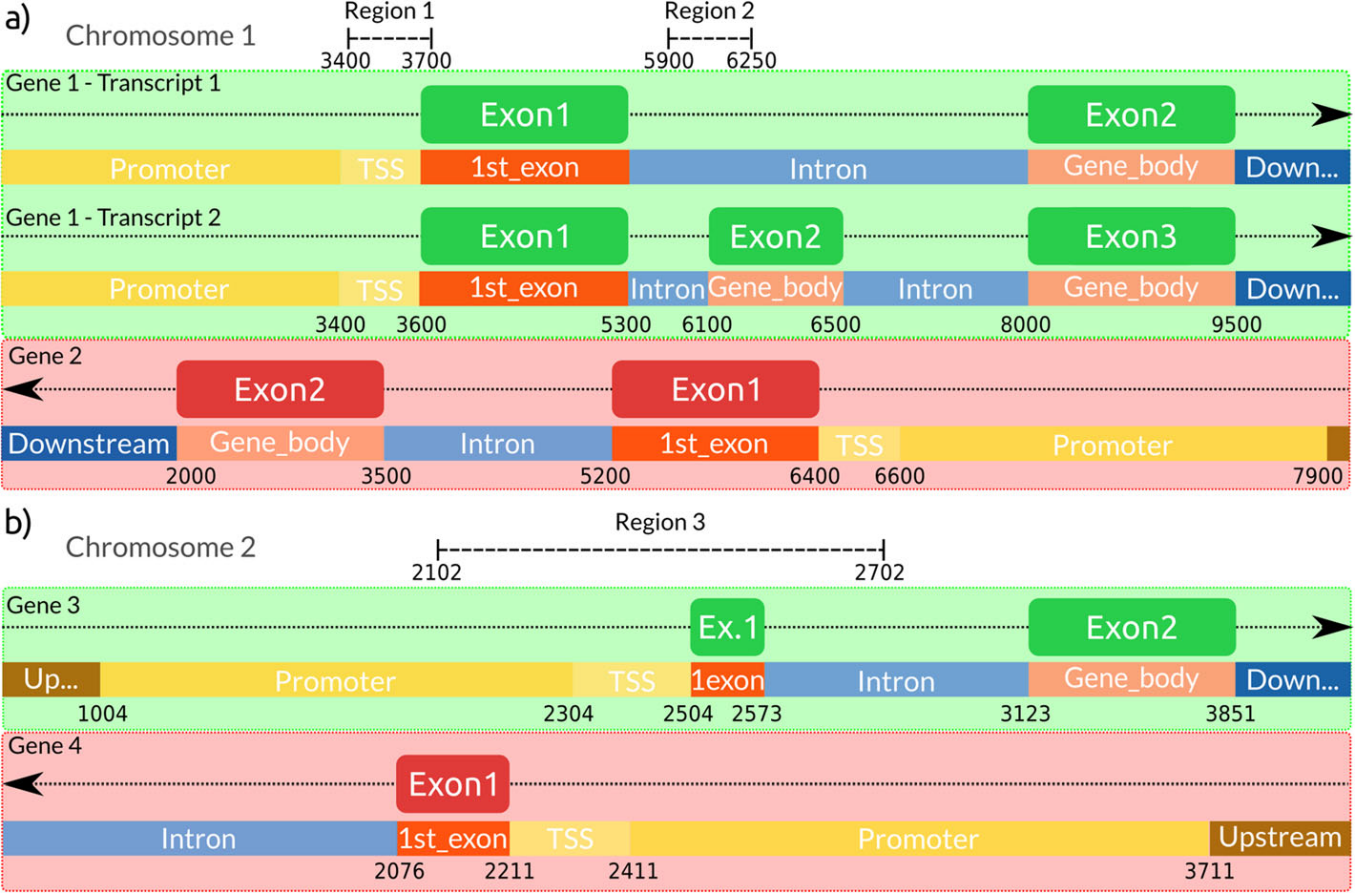
Examples of two different situations that would result in a region being associated with more than one gene. **a** Two overlapped genes with different isoforms. **b** Two different genes with common areas overlapping the region (quasi-overlapping genes)

There are two different cases in which a region could be associated with more than one gene: when two or more genes overlap (Fig. 2a) or when two (or more) genes are so close (“quasi-overlapping” genes) that the region falls in the overlapping areas of the two genes (Fig. 2b).

**Fig. 3:**
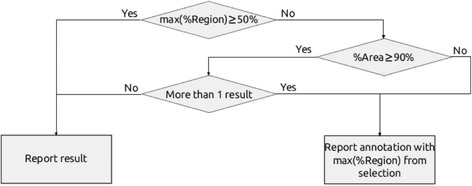
Flowchart describing the rules used by RGmatch to decide the gene area to annotate the region-transcript association (default algorithm options)

When the region overlaps several areas of a gene but the user needs to choose a single area per gene or transcript to annotate the association, a set of rules has to be defined in order to select the most appropriate one. The rules defined by RGmatch are based on the percentage of the region overlapping each area of the gene (“*PercRegion”*), the percentage of each gene area that is overlapped by the region (“*PercArea*”), and a rank of priorities for the areas to be used in the case of any ties (by default: TSS, 1st EXON, PROMOTER, TTS, INTRON, GENE BODY, UPSTREAM, DOWNSTREAM). As summarized in Fig. 3, if there is an area for which *PercRegion ≥ w* (50 % by default), this area will be the annotation for that region-transcript association. Otherwise, the algorithm uses the area with *PercArea ≥ v* (90 % by default).

When several areas meet this condition, the one with highest *PercRegion* is selected. In the case of ties, the selected area is determined according to the list of priorities. The default percentages to apply the rules (*v* and *w*) and the default area priorities can be easily modified by the user.

One of the main advantages of RGmatch is its ability to report the associations at different aggregation levels (exon, transcript, or gene). By default, it reports all possible associations to the different areas of the exons. When choosing the report at the ‘transcript aggregation level’, the algorithm applies the set of previously-defined rules in order to return a single area per region and transcript. The same rules apply when reporting at the ‘gene aggregation level’, but in this case, if the region is located in different areas for each transcript of a given gene, the rank of priorities will be used to annotate the association to only one of them.

RGmatch generates a tabular text output file with the following columns:

- **Region**: Identifier (ID) of the region being associated. This ID is generated by RGmatch and consists of the chromosome, start, and end position, separated by an underscore (chr_start_end).
- **Midpoint**: Midpoint of the region being associated.
- **Gene**: Gene ID for the gene that has been associated to the region.
- **Transcript**: Transcript ID for the transcript that has been associated to the region. When reporting at the gene aggregation level the algorithm will report all the possible transcripts in the case of internal ties.
- **Exon**: Exon number of the exon associated to the region. In the case of transcript ties, when reporting at gene aggregation level, the value reported will be -1.
- **Area**: Area of the gene (or transcript) where the region falls.
- **Distance**: Distance from the TSS or TTS to the midpoint of the region. When the region overlaps a gene, the distance reported is 0.
- **PercRegion**: Percentage of the region that overlaps the area of the gene reported.
- **PercArea**: Percentage of the reported area overlapped by the region.
- If the input BED file had more columns than the three mandatory ones, these columns are attached in the output file after the *PercArea* column.

The associations rendered by RGmatchat the three different aggregation levels for the two examples shown in Fig. 2, according to the rules described and using the default parameters, are shown in Table 1, and to illustrate how the algorithm works some of them are also described below.

**Table 1:** Table showing the results at the exon level for the example shown in Fig. 2

Region	Midpoint	Gene	Transcript	Exon	Area	Distance	PercRegion	PercArea
1_3400_3700	3550	Gene2	Tr1_Gene2	2	INTRON	0	66.45	−1
1_3400_3700	3550	Gene2	Tr1_Gene2	2	GENE_BODY	0	33.55	6.73
1_3400_3700	3550	Gene1	Tr1_Gene1	1	TSS	0	66.45	100.0
1_3400_3700	3550	Gene1	Tr1_Gene1	1	1st_EXON	0	33.55	5.94
1_3400_3700	3550	Gene1	Tr2_Gene1	1	TSS	0	66.45	100.0
1_3400_3700	3550	Gene1	Tr2_Gene1	1	1st_EXON	0	33.55	5.94
1_5900_6250	6075	Gene2	Tr1_Gene2	1	1st_EXON	0	100	29.23
1_5900_6250	6075	Gene1	Tr2_Gene1	2	INTRON	0	56.98	−1
1_5900_6250	6075	Gene1	Tr2_Gene1	2	GENE_BODY	0	43.02	37.66
2_2102_2702	2402	Gene4	Tr1_Gene4	1	TSS	0	33.28	100.0
2_2102_2702	2402	Gene4	Tr1_Gene4	1	PROMOTER	0	48.42	22.38
2_2102_2702	2402	Gene4	Tr1_Gene4	1	1st_EXON	0	18.30	80.88
2_2102_2702	2402	Gene3	Tr1_Gene3	1	TSS	0	33.28	100.0
2_2102_2702	2402	Gene3	Tr1_Gene3	1	PROMOTER	0	33.61	15.54
2_2102_2702	2402	Gene3	Tr1_Gene3	1	1st_EXON	0	11.65	100
2_2102_2702	2402	Gene3	Tr1_Gene3	1	INTRON	0	21.46	−1

Region 1 (1_3400_3700) from Fig. 2a overlaps Gene 1 and Gene 2. Gene 1 has two different transcripts. If we report at the exon level, RGmatch returns all the areas of the different genes overlapped by the region. In this example, Region 1 overlaps the entire ‘TSS’ (100 %) and part of the ‘1st_exon’ (5.94 %) of both transcripts of Gene 1, and part of the ‘gene_body’ and ‘intron’ areas of Gene 2. RGmatch reports the different overlap percentages, except for introns (for which it returns a -1 result). Of the total length of Region 1, 66 % overlaps the ‘TSS’ of Gene 1 (for both transcripts) and the ‘intron’ of Gene 2. According to the previously described rules, given that this percentage is higher than the 50 % set as the threshold, these areas will be returned when reporting at the transcript level. In the gene-level report, both Gene1 and Gene2 are associated with Region 1 (overlapping genes). For Gene1, the association is annotated to ‘TSS’ since both transcripts had the same annotation.

Region 3 from Fig. 2b overlaps Gene 3 and Gene 4, and has a percentage of overlap of 33.28, 33.61, 11.65, and 21.46 % with the ‘TSS’, ‘promoter’, ‘1st_exon’, and ‘intron’ regions of Gene 3, respectively. When reporting at the transcript or gene aggregation levels, since these overlap percentages do not exceed 50 % in any case, we have to look at the percentage of each gene area overlapped by the region. Two different areas (‘TSS’ and ‘1st_exon’) are completely overlapped with a percentage higher than 90 %, and so they are tied. In this case the algorithm returns the area with the highest percentage of the region overlapping it, which corresponds to the TSS (33.28 %). The same procedure also has to be applied to Gene 4, this process results in the same TSS annotation. Therefore, Region 3 will have two associated genes reported with the ‘TSS’ annotation (quasi-overlapping genes).

RGmatch provides many configuration options and the user can modify the priorities and rules followed to associate a region with a gene area. The following arguments can be optionally set by the user:

- **Report**: Argument to select the aggregation level for the report. By default, it is set to ‘exon’ and all possible associations to all the different areas of a gene or genes where the region overlaps will be reported. When it is set to ‘transcript’ or ‘gene’ the rules explained above are applied.
- **Distance**: By default, a region will be associated with a gene if it is closer than 10 kbp.
- **TSS**: Area starting at the transcription start site of a gene and finishing *t* bp upstream from that point. By default, *t* = 200.
- **TTS**: Intergenic area starting at the transcription termination site of a gene with a length of *s* bp. By default, *s* = 0, so this area is not considered unless this parameter is modified by the user.
- **Promoter**: Area starting one nucleotide after the predefined TSS area and extending up to *p* bp upstream from that point. By default, *p* = 1300.
- **PercArea**: Threshold for the percentage of the gene area overlapped by the region, used in the selection rules (see flowchart in Fig. 3). By default, this is set at 90 %.
- **PercRegion**: Threshold for the percentage of the region overlapping the gene area, used in the selection rules (see flowchart in Fig. 3). By default, this is set at 50 %.
- **Rules**: In case of ties after following the rules shown in Fig. 3, the algorithm will decide the area to annotate the association to according to a rank of priorities, by default this is: TSS, 1st_EXON, PROMOTER, TTS, INTRON, GENE_BODY, UPSTREAM, and DOWNSTREAM. To modify these priorities, a string containing the eight disjoint areas must be introduced.
- **Gene**: Tag indicating which gene identifier from the GTF annotation file is to be reported. By default ‘gene_id’ is used.
- **Transcript**: Tag indicating which transcript identifier from the GTF annotation file is to be reported. By default ‘transcript_id’ is used.
- **GTF**: Mandatory input. GTF annotation file. Files compressed with gzip are also accepted.
- **BED**: Mandatory input. BED file with the set of genomic regions to be matched. Files compressed with gzip are also accepted.
- **Output**: Mandatory input. Full path and name of the file where the output will be written.

## Results and discussion

In order to show the functionalities and main advantages of RGmatch, we compared it to the other methods available: HOMER, GREAT, CisGenome, Seq2pathway, and ChIPseeker. Comparisons are difficult because, on the one hand, there is no gold-standard data set of true associations between the genomic regions and the genes and, on the other hand, the goal of the different methods is not always exactly the same. For instance, GREAT and Seq2pathway do not only return the closest gene but also other distal genes by following an approach that is completely different to the other methods. GREAT assigns a ‘regulatory domain’ for each gene, so if any region lies within the regulatory domain, it is assumed to regulate the gene. There are three options to define this regulatory domain. The default option (the one we compared RGmatch to), called the ‘basal plus extension’, assigns a ‘basal regulatory region’ that extends 5 kbp upstream and 1 kbp downstream of the TSS, irrespective of the presence of any neighboring genes. Based on a similar approach, Seq2pathway takes the functional impact of coding and non-coding genes into account to make associations. In the following sections we provide both qualitative and quantitative comparisons based on the results obtained with a publicly available set of genomic regions.

### Qualitative comparison to the state of the art methods

In this section, we highlight the characteristics of RGmatch that make it different from any of the other approaches (see a summary in Table 2), and which therefore support the need to make this novel tool available to the research community.

**Table 2:** Comparison of the functionalities of the different algorithms

	RGmatch	Homer	GREAT	CisGenome	Seq2pathway	ChIPseeker
User − friendly	Command line	Command line	Web tool	Command line/GI (only in Windows)	R/Bioc	R/Bioc
Adaptable to pipelines	Yes	Yes^a^	No	Yes^a^	Yes^a^	Yes^a^
Input format	BED (also gzip-compressed BED file)	BED	BED (only 3 columns)	BED - > COD	BED - > GRanges	BED
Association resolution	Gene, transcript, exon	Gene, transcript	Gene	Gene	Gene	Gene, transcript
Area annotation	Yes	Yes	No	No	Yes	Yes
Flexibility	Distance, Areas, Rules, Area priorities	No	Distance	Distance	Search radius	Area priorities, TSS distance
Supported species	All	All	3	12	2	All^b^
Output: Gene IDs?	Any in the GTF	Gene and transcript IDs	Gene names	Gene IDs	Gene IDs and gene names	Gene and transcript IDs
Output: Distance?	Yes	Yes	Yes	No	Yes	Yes
Output: Overlapping genes?	Yes	No	No	No	Yes	No

^a^HOMER and CisGenome can be integrated in analysis pipelines, although the process to obtain the annotations and parse these results is not as straightforward as with RGmatch. Seq2pathway and ChIPseeker can also be integrated with additional scripting

^b^It supports all species, provided the input format is a TxDb R object. This format can be obtained from a GTF file by using the makeTxDbFromGFF function in the GenomicFeatures package

#### User-friendly

RGmatch and HOMER are easy-to-use command line algorithms that can be run locally on any computer and in any operating system provided Python or Perl interpreters are installed. GREAT is accessible via their website, which makes it user-friendly on any operating system, but it cannot be used locally. CisGenome can also be used in any operating system via command line and has a graphical interface, but only for Windows. On the contrary, ChIPseeker and Seq2pathway are both R packages that can be easily used if the R interpreter is installed. However, we had problems using Seq2pathway on the Linux platform because the association function did not work.

#### Adaptable to pipelines

All methods except GREAT, which is a web tool, can be easily integrated into any analysis pipeline. HOMER is a suite of tools, and the whole suite has to be installed for the method to work. As for all R packages, ChIPSeeker and Seq2pathway, can also be integrated into any analysis pipeline, although some additional scripting is required. In contrast, RGmatch can be directly used in any pipeline and does not require additional steps or modules to work.

#### Input format

RGmatch, GREAT, HOMER, and ChIPSeeker take a BED file containing the regions to be associated as input. CisGenome and Seq2pathway require the BED file to be converted into their own formats. GREAT accepts a 3-column BED file. The other methods accept BED files containing information other than genome coordinates, but only RGmatch and ChIPSeeker return the additional columns in the output file.

#### Association resolution

A unique feature of RGmatch is its ability to report associations at the exon, transcript, or gene level. GREAT, CisGenome, and Seq2pathway only report associations at the gene level, whereas HOMER and ChIPSeeker can report associations at the gene or transcript level.

#### Location of the region

RGmatch, HOMER, Seq2pathway, and ChIPSeeker report the area of the gene where the region overlaps for each association. Neither GREAT nor CisGenome return this information.

#### Flexibility

RGmatch, CisGenome, Seq2pathway, and GREAT let users modify the basic parameters (related to the maximum distance) used to associate a region to a gene. HOMER, on the contrary, always associates the region to a gene no matter how far it is. RGmatch and ChIPSeeker also allow the user to modify the length of some gene areas, as well as the priorities for annotating the association with the gene area. In addition, RGmatch offers a flexible definition of the association rules, while this is not possible in HOMER or Seq2pathway.

#### Supported species

RGmatch, HOMER, and ChIPseeker work with any organism as long as the user provides the GTF annotation file. However, the annotations must be converted to TxDb R objects for ChIPseeker to function. GREAT, Seq2pathway, and CisGenome only work with the species list they provide; at the moment, GREAT and Seq2pathway support four species assemblies each, (both support hg19, mm9, and mm10, plusdanRer7andhg38 in GREAT and Seq2pathway, respectively), and CisGenome supports 12 different species.

#### Output

All of the algorithms compared return a tabulated file containing the region-gene associations and some additional information. Only RGmatch and ChIPSeeker preserve the original columns in the BED file when more than the three mandatory columns containing the genomic positions are provided (e.g. coverage, quality, *p*-values, etc. may also be included in the region BED file). RGmatch also allows the user to choose the gene identifier to be reported among all the identifiers in the GTF file. In HOMER and ChIPseeker, the user can choose between gene and transcript IDs, CisGenome reports the gene ID, and GREAT returns gene names. All the methods except CisGenome report the distance between the gene and the region. RGmatch, HOMER, ChIPseeker, and Seq2pathway return the area of the gene overlapped by the region. The gene area definitions are similar forHOMER, ChIPseeker, and RGmatch, or at least they can be made almost equivalent by tuning the RGmatch parameters. However, the column containing the gene area in the HOMER and ChIPseeker outputs also contains additional information so this column cannot be directly used in further analyses where a categorical classification of the gene areas is needed (see output examples in Additional file 1). Another unique feature of RGmatch and Seq2pathway is that if a region can be associated with two or more overlapping genes, all of them are reported as different rows in the output file, while the other methods only provide one associated gene in these cases.

### Quantitative comparison

To quantitatively assess the functionality of our approach, we compared RGmatch to HOMER and CisGenome using a public set of genomic regions. We discarded GREAT and Seq2pathway from the comparison because they follow a completely different approach to associate chromatin regions, meaning that the results are not directly comparable. We also decided not to include ChIPseeker because it is very similar to HOMER. The public set of genomic regions, containing 2638 regions, comes from a human ChIP-Seq experiment, and was downloaded from the Sequence Read Archive (SRA) with accession number GSE55727. The annotation (GTF file) was downloaded from Ensembl GRCh37.75.

In order to make the outputs comparable between the methods, the RGmatch report was performed at the gene aggregation level, the maximum distance for reporting associations was set to 1000 kbp to allow at least one association per region, the promoter length was set to 0, and the TSS area was set to 1kbp. The rest of the parameters were left at their default values. We used the default parameters for HOMER. To run CisGenome, first the GTF was converted to refFlat format using the gtfToGenePred tool from the University of California Santa Cruz Genomics Institute, and then the BED file was converted to COD format using the file_bed2cod tool provided by CisGenome. CisGenome was then run setting the distance limits to 1000 kbp and leaving the rest of the parameters at their default values. Regions corresponding to chromosomes X and Y were removed from the BED file used for all of the algorithms because CisGenome does not take them into account, which left a total of 2592 regions.

**Fig. 4:**
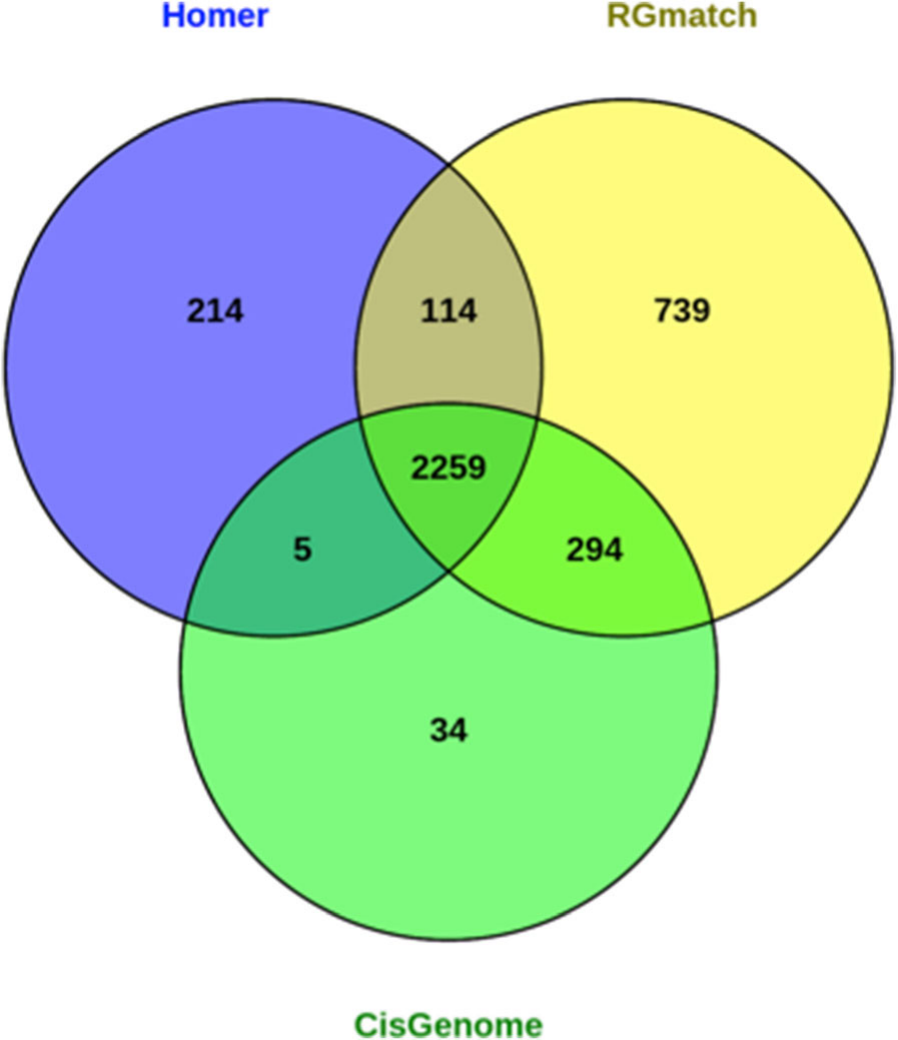
Venn diagram showing the number of region-gene associations obtained with the HOMER, RGmatch, and CisGenome methods

Each of the final 2592 regions was associated with a single gene by HOMER and CisGenome. RGmatch returned 3406 associations due to overlapping and quasi-overlapping genes. The percentage of common associations reported by the three methods was high (Fig. 4). Almost 100 % of the associations called by RGmatch were also reported by HOMER and/or CisGenome. However, RGmatch reported 739 associations that were not called by the other two methods. Most of them (731) were due to the fact that RGmatch can associate regions to two different genes, so one of the two genes is reported by the other two methods, but the second gene is only reported by RGmatch. The reason for the remaining 8 associations, that were exclusively detected by RGmatch, was because RGmatch associated the region to the closest gene (which was downstream), while HOMER associated it to a more distal gene in an upstream area. There is no clear reason why CisGenome returned a different association for these cases. The associations that were common to RGmatch and only one of the other two methods were generally also due to RGmatch associating the region to two overlapping (or quasi-overlapping) genes whereas HOMER reported one of the two associations and CisGenome reported the other.

We also observed that, in some cases where the methods returned different results, the associated region was far away from the genes. RGmatch associated the region to the closest gene, even if the region was downstream from the gene. In these cases, CisGenome tends to associate the region to a gene with an upstream annotation (even if it is not the closest gene), while HOMER either does the same or chooses a downstream annotation but to the second closest gene.

RGmatch and HOMER also report the area of the gene where the region overlaps. However, the definition of the gene areas reported by these two methods is not exactly the same. HOMER defines their ‘promoter-TSS’ as the region comprising −1kbp to +100 bp and the ‘TTS’ as from −100 bp to +1kbp. In order to cover the same areas, we defined our ‘TSS’ area as −1kbp to −1 bp and removed the ‘promoter’ area. This way, HOMER’s TSS area was equivalent to ours plus the first 100 bps from our ‘1st_exon’ area, and our ‘Downstream’ area was equivalent to Homer’s TTS and Intergenic area, etc. (see all the equivalences in Table 3).

**Table 3:** Equivalences between the gene areas defined by RGmatch and HOMER

RGmatch	HOMER
INTRON	Intron
UPSTREAM	Intergenic
DOWNSTREAM	TTS; Intergenic
GENE_BODY	exon; 3′ UTR; 5′ UTR
TSS	promoter-TSS
1st_EXON	exon; promoter-TSS; 5′ UTR; 3′ UTR

Table 4 shows the number of associations reported by HOMER and RGmatch with equivalent annotations for the region location (in green), accounting for the vast majority (more than 95 % of the reported associations). Associations where the gene area did not agree are indicated in red. Discrepancies are due to regions overlapping several areas of the gene. In such cases, the true location of the region in the gene is unclear. While HOMER chooses the area overlapping the midpoint of the region, the RGmatch annotation is based on the overlap percentage and on the priorities chosen by the user, allowing them to fine-tune the association results depending on their analysis goals.

**Table 4:**
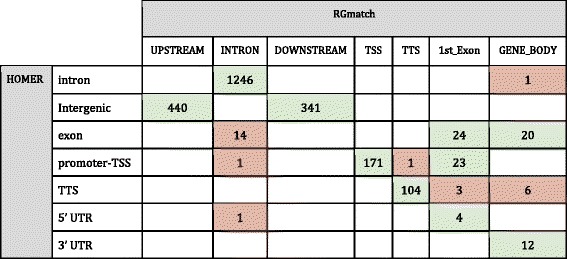
Annotations for the region location within the gene returned by RGmatch (columns) and HOMER (rows)

Associations with equal or equivalent annotations in both methods are shown in green, and associations with different annotations are shown in red

In summary, the association results from RGmatch are comparable to the results provided by other methods. Nevertheless, RGmatch is more flexible than other approaches because it allows the rules used to compute the associations, and annotate them with the region location within the gene, to be defined by the user. Moreover, it returns all the possible associations when the region overlaps more than one gene (overlapping or quasi-overlapping genes), and the output is easier for the user to understand and re-use.

To check the efficiency of the algorithms, we compared the computation time and memory used when running the algorithms on the full human ChIP-seq example (2638 regions, including the X and Y chromosomes) with the human reference genome annotation GTF file. RGmatch took 32 s to obtain the results and required 1 GB of RAM memory. In contrast, HOMER took 1 min and 30 s and required up to 3 GB of RAM. CisGenome was almost instantaneous, since some prior extra work had been performed. These calculations were performed on an Intel(R) Xeon(R) CPU E3-1225 V2 @ 3.20GHz machine.

RGmatch has been designed in order to check only the proximal annotations for each region. This implies that it is highly scalable despite having a large number of regions. In our tests, RGmatch obtained results in 15 s using a file with ~25,000 regions, 50 s with ~200,000 regions and 122 s with ~600,000 regions in a 2.4 GHz Intel Core i5. The slowest step is the internal ordering of the regions and annotations, but the association step is really straightforward.

## Conclusions

As sequencing technologies evolve and studies that integrate gene expression with chromatin features become more common, the need to associate genomic regions to genes in order to understand regulatory mechanisms has increased. Although there are a number of publicly-available tools to perform this task, most of them have limitations in terms of flexibility or usability.

In this work, we present RGmatch, a user-friendly tool for matching genomic regions and genes (as well as transcripts or exons), which reports the area of the gene where the region overlaps. RGmatch supports all species as long as the user provides the GTF file with the reference genome annotation. The tool is a freely accessible Python script, which promotes integration into broader analysis pipelines. RGmatch is a valuable resource for facilitating analysis in multi-omics experiments involving gene expression and different types of chromatin features.

The main advantages of RGmatch, when compared to the state-of-the-art methods, are its flexibility for the user to define its association rules, gene areas, gene identifiers to be reported, and priorities for the gene area annotation when the region overlaps different areas of the gene, as well as its ability to report associations at different aggregation levels. In addition, when a genomic region overlaps several genes, all the associations are returned. Hence RGmatch provides a biologically meaningful set of rules and parameters that can be tuned by users to adapt the associations to their preferences or needs.

## Additional file

Additional file 1: Examples of the output files for some of the compared algorithms. (DOCX 20 kb)

## Abbreviations

ATAC-seq: Assay for Transposase-Accessible Chromatin with high-throughput sequencing
ChIP-seq: Chromatin immune precipitation sequencing
DNase-seq: DNase I hypersensitive sites sequencing
GREAT: Genomic regions enrichment of annotations tool
HOMER: Hypergeometric Optimization of Motif EnRichment
ID: Identifier
Methyl-seq: Methylation sequencing
SRA: Sequence Read Archive
TSS: Transcription start site
TTS: Transcription termination site
