# Supplementary material for: RGmatch: matching genomic regions to proximal genes in omics data integration
Source: BMC Bioinformatics. 2016 Nov 22;17(Suppl 15):1–10. doi: 10.1186/s12859-016-1293-1 (PMC5133492; doi:10.1186/s12859-016-1293-1)
Supplement: Additional file 1: — Examples of the output files for some of the compared algorithms. (DOCX 20 kb) [file 12859_2016_1293_MOESM1_ESM.docx]

Additional file 1

**OUTPUT: Area annotation**

Tables 1-3 are an extract of the output given by RGmatch, ChIPseeker and Homer respectively. Figure 1 shows the genomic surroundings of the first region shown in the tables (chr1: 1112397-1112713). The columns corresponding to the gene area annotation in ChIPseeker and Homer include more detailed information than simply the annotated tag. However, using these columns for further analysis is not so easy to parse as in RGmatch, where only the tag is given. Moreover, the additional information given by the other two methods is usually related to other nearby or overlapping genes butRGmatch also returns this information whenever it is valuable but in different rows of the file.

| Region | Midpoint | Gene | Transcript | Exon/Intron | Area | Distance | PercRegion | PercArea |
| --- | --- | --- | --- | --- | --- | --- | --- | --- |
| 1_1112397_1112713 | 1112555 | ENSG00000162571 | ENST00000379290,ENST00000379289 | 3,3 | INTRON | 0 | 100 | 6.746 |
| 1_1112397_1112713 | 1112555 | ENSG00000205231 | ENST00000379317 | 1 | INTRON | 0 | 100 | 10.68 |
| 1_1241942_1242434 | 1242188 | ENSG00000131584 | ENST00000492936 | 1 | TSS | 879 | 74.65 | 36.8 |
| 1_1838807_1839250 | 1839028 | ENSG00000169885 | ENST00000307786 | 1 | UPSTREAM | 7238 | 100 | -1 |
| 1_3227232_3227670 | 3227451 | ENSG00000272235 | ENST00000607061 | 1 | TSS | 791 | 97.82 | 42.9 |
| 1_3227232_3227670 | 3227451 | ENSG00000142611 | ENST00000514189,ENST00000512462,ENST00000378398,ENST00000511072,ENST00000441472,ENST00000378391,ENST00000270722,ENST00000442529,ENST00000463591 | 3,2,3,3,3,3,3,3,1 | INTRON | 0 | 100 | 0.31 |
| 1_4590977_4591471 | 4591224 | ENSG00000227169 | ENST00000438791 | 2 | DOWNSTREAM | 20571 | 100 | -1 |

Table 1.RGmatch real output example. Area annotation is in column “Area”.

| **Seqnames** | **Ranges** | **Strand** | **Annotation** | **GeneChr** | **geneStart** | **geneEnd** | **geneLength** | **geneStrand** | **geneID** | **TranscriptID** | **distancetoTSS** |
| --- | --- | --- | --- | --- | --- | --- | --- | --- | --- | --- | --- |
| 1 | [1112397, 1112713] | * | Intron (ENST00000379290/ENSG00000162571, intron 3 of 15) | 1 | 1108436 | 1114935 | 6500 | - | ENSG00000205231 | ENST00000379317 | 2538 |
| 1 | [1241942, 1242434] | * | Intron (ENST00000354700/ENSG00000131584, intron 23 of 23) | 1 | 1227770 | 1241309 | 13540 | - | ENSG00000131584 | ENST00000492936 | -1125 |
| 1 | [1838807, 1839250] | * | Distal Intergenic | 1 | 1846266 | 1848733 | 2468 | + | ENSG00000169885 | ENST00000307786 | -7459 |
| 1 | [3227232, 3227670] | * | Intron (ENST00000511072/ENSG00000142611, intron 3 of 15) | 1 | 3223200 | 3226660 | 3461 | - | ENSG00000272235 | ENST00000607061 | -1010 |
| 1 | [4590977, 4591471] | * | Distal Intergenic | 1 | 4611795 | 4612205 | 411 | - | ENSG00000227169 | ENST00000438791 | 21228 |

Table 2. ChIPseeker real output example. Area annotation is in column “Annotation”.

| **PeakID** | **Chr** | **Start** | **End** | **Strand** | **Peak Score** | **Focus Ratio/Region Size Annotation** | **DetailedAnnotation** | **Distance to TSS** | **NearestPromoter ID** |  |
| --- | --- | --- | --- | --- | --- | --- | --- | --- | --- | --- |
| 595 | 1 | 1112398 | 1112713 | + | 0 | NA | intron (ENSG00000205231, intron 1 of 1) | 2380 | ENSG00000205231 |  |
| 1033 | 1 | 1241943 | 1242434 | + | 0 | NA | promoter-TSS (ENSG00000131584) | -1759 | ENSG00000169972 |  |
| 1813 | 1 | 1838808 | 1839250 | + | 0 | NA | Intergenic | -7518 | ENSG00000169885 |  |
| 2634 | 1 | 3227233 | 3227670 | + | 0 | NA | promoter-TSS (ENSG00000272235) | -791 | ENSG00000272235 |  |
| 62 | 1 | 4590978 | 4591471 | + | 0 | NA | Intergenic | 20981 | ENSG00000227169 |  |

Table 3. Homer real output example. Area annotation is in column “DetailedAnnotation”.
